# Regulatory loop between the CsrA system and NhaR, a high salt/high pH regulator

**DOI:** 10.1371/journal.pone.0209554

**Published:** 2018-12-27

**Authors:** Jarosław E. Król

**Affiliations:** Department of Microbiology and Cell Science, University of Florida, Gainesville, Florida, United States of America; University of Houston, UNITED STATES

## Abstract

In *E*. *coli*, under high pH/high salt conditions, a major Na^+^/H^+^ antiporter (NhaA) is activated to maintain an internal pH level. Its expression is induced by a specific regulator NhaR, which is also responsible for *osmC* and *pgaA* regulation. Here I report that the NhaR regulator affects the carbon storage regulatory Csr system. I found that the expression of all major components of the Csr system–CsrA regulator, CsrB and CsrC small RNAs, and the CsrB and CsrC stability were indirectly affected by *nhaR* mutation under stress conditions. Using a combination of experimental and *in silico* analyses, I concluded that the mechanism of regulation included direct and indirect activation of a two-component system (TCS) response regulator–UvrY. NhaR regulation involved interactions with the regulators H-NS and SdiA and was affected by a naturally occurring spontaneous IS*5* insertion in the promoter region. A regulatory circuit was proposed and discussed.

## Introduction

Living organisms have evolved many genetic mechanisms to deal with different stressful environmental factors. Bacterial pH homeostasis is important for physiology, ecology, and pathogenesis. The effect of low pH on bacterial physiology has been subjected to many studies mostly because an acidic environment is more prevalent in nature [1–5]. Lower pH also plays an important role in human defense systems, starting from the lower pH of the skin and vagina, to the highly acidic stomach environment, and ending with the low pH inside lysosomes in macrophages [6, 7]. High pH environments include natural habitats such as alkaline soda lakes and highly alkaline segments of the hindgut of certain insects, as well as industrial settings such as indigo dye plants, sewage plants, and geochemically unusual groundwater with pH values of >12 [8]. Resistance to base may also enhance survival of bacteria as they pass through the pylorus and enter the upper intestine, where they encounter alkaline pancreatic secretion [9].

Under alkaline and osmotic stress, living cells must maintain an externally directed sodium gradient and a relatively constant intracellular pH [10]. Membrane proteins that exchange Na^+^ (or Li^+^) for H^+^, called Na^+^/H^+^ antiporters, play important roles in these processes. NhaA is the key antiporter that protects *E*. *coli* against sodium stress, and it is essential for growth in the presence of high sodium concentrations. NhaB, a second antiporter, is necessary only in NhaA mutants [11, 12]. The *nhaA* gene is located in a two-gene operon, *nhaAR*, where the *nhaR* gene encodes a LysR-OxyR family transcriptional regulator [13]. The *nhaAR* operon is induced by the presence of monovalent cations, high pH, low temperature, and stationary phase [14, 15]. Its expression is driven by two promoters. P1 is an NhaR-dependent, Na^+^-induced, and H-NS-affected promoter in both the exponential and stationary phases. P2 shows minimal activity during the exponential phase but is somehow induced in the stationary phase and becomes the major promoter. P2 is activated by sigma(S) and is shown to be nonresponsive to Na^+^, as well as NhaR and H-NS activities [16, 17]. Recent results showed that *nhaR* is postranscriptionally regulated by the CsrA protein [18]. The expression of *nhaAR* genes is repressed by the H-NS in the lack of stress; however, in the presence of Na^+^, NhaR functions as a positive regulator of *nhaA* and overcomes the repressive effects of H-NS [17]. NhaR also activates the transcription of *osmC*, which is required for resistance to organic peroxides and long-term survival in the stationary phase [19, 20], as well as the *pgaABCD* operon required for biofilm formation in *E*. *coli* [21, 22]. H-NS is a global regulator [23] that is induced at low temperature and shown to be a common regulator of multiple iron and other nutrient acquisition systems preferentially expressed at 37°C, as well as general stress response, biofilm formation, and cold shock genes highly expressed at 23°C [24].

Biofilms are associated with various kinds of stress protection, including pH resistance. Biofilm formation is a complicated, multistage process, which is affected by many regulatory systems (see[25]). The RNA-binding protein CsrA plays an important role in many biological processes including quorum sensing and biofilm formation [26]. CsrA is a global regulatory protein that interacts with target mRNAs, changing their stability and/or translation. CsrA activates genes that are necessary for bacterial growth or are expressed in the exponential phase of growth and inhibits genes expressed in stationary phase or encoding secondary metabolites. CsrA inhibits biofilm formation by a posttranscriptional repression of *pgaABCD*, responsible for synthesis of a polysaccharide adhesin poly-beta-1,6-N-acetyl-d-glucosamine [27]. On the other hand, CsrA activates motility by increasing stability of *flhDC* mRNA [28]. CsrA expression is increased during entrance into stationary phase. Expression of the *csrA* gene depends on CsrA activity in the cell [29]. That activity is controlled by two small noncoding RNAs, CsrB and CsrC, which bind to and sequester multiple copies of CsrA [30, 31]. Expression of these small RNAs is affected by the SOS system [32] and by a two-component system (TCS) regulator UvrY [33]. The BarA/UvrY system is activated by an increased pH [34] as well as quorum-sensing regulator SdiA [33]. As SdiA activity is also postransciptionally regulated by the CsrA protein [35], the whole system forms a closed regulatory circuit. A recent paper by Camacho et. al. [36] demonstrated that CsrA is indirectly required for proper *uvrY* expression and also for activation of the BarA kinase activity.

In this paper, I examined regulatory interactions of the specific NhaR regulator and the CsrA circuit. I was able to show that under some environmental conditions NhaR is necessary for the activity of the CsrA system. Conducted experiments indicated that NhaR indirectly affects expression of *csrA*, *csrB*, and *csrC* and the stability of these small RNAs. A *nhaR* mutation directly and indirectly affects expression of *uvrY* genes. In the case of *uvrY*, the mode of regulation seems to be similar to that proposed for the *nhaAR* operon where NhaR is necessary to overcome H-NS inhibition. I found that naturally occurring transposition of the IS*5* into the *uvrY* promoter region abolishes regulation of the *uvrY* gene and affects the whole CsrA circuit. I present and discuss a model of complex interactions between NhaR and the CsrA system.

## Materials and methods

### Bacterial strains and growth conditions

The *E*. *coli* strains, plasmids, and bacteriophage used in this study are listed in Table 1. All mutations except Δ*H-NS* were transferred into the JEK710 strain from KEIO collection strains by P1 transduction. JEK1553Δ*H-NS* was constructed by the λRed system and pKD3 plasmid [37]. Bacteria were grown at 37°C or 26°C, shaking at 250 rpm, in Luria-Bertani (LB) medium. LB broth pH 8.4 stabilized with 0.1 mM 3-[N-tris(hydroxymethyl)methylamino]propanesulfonic acid (TAPS) was used for the *nhaR* gene effect tests. Media were supplemented with antibiotics, as needed, at the following concentrations: kanamycin: 50 μg/ml; gentamicin: 10 μg/ml; ampicillin: 100 μg/ml; chloramphenicol: 25 μg/ml; and tetracycline: 10 μg/ml.

**Table 1:** Bacterial strains, plasmids and bacteriophage used in this study.

Strain / plasmid/bacteriophage	Genotype	Reference
*E*. *coli* EC100	*F*^*-*^ *mcrA Δ(mrr-hsdRMS-mcrBC) Φ80dlacZΔM15 ΔlacX74 recA1 endA1 araD139 Δ(ara*, *leu)7697 galU galK λ*^*-*^ *rpsL (Str*^*R*^*) nupG*	Epicentre
*E*. *coli* K12 MG1655	F- λ-	ATCC
GS1114	CF7787 *Δ*(*λatt-lom*)::*bla Φ*(*csrC-lacZ*)*1*(*hyb*) Amp^R^	[31]
KSB837	CF7787 *Δ*(*λatt-lom*)::*blaΦ*(*csrB-lacZ*) *1*(*hyb*) Amp^R^	[31]
KSA712	CF7787 *Δ*(*λatt-lom*)::*blaΦ*(*csrA’-‘lacZ*) *1*(*hyb*) Amp^R^	[39]
KSY009	CF7787 *Δ*(*λatt-lom*)::*blaΦ*(*uvrY’-‘lacZ*) *1*(*hyb*) Amp^R^	[33]
JEK710	MG1655 LacZ^-^ Gm^R^	This study
JEK736	JEK710 Δ*nhaR* Gm^R^Km^R^	This study
JEK821	JEK710 *Δ*(*λatt-lom*)::*bla Φ*(*csrC-lacZ*)*1*(*hyb*) Amp^R^Gm^R^	This study
JEK825	JEK710 *Δ*(*λatt-lom*)::*bla Φ*(*csrB-lacZ*)*1*(*hyb*) Amp^R^Gm^R^	This study
JEK829	JEK736 *Δ*(*λatt-lom*)::*bla Φ*(*csrC-lacZ*)*1*(*hyb*) Amp^R^Gm^R^Km^R^	This study
JEK833	JEK736 *Δ*(*λatt-lom*)::*bla Φ*(*csrB-lacZ*)*1*(*hyb*) Amp^R^Gm^R^Km^R^	This study
JEK879	JEK710 *Δ*(*λatt-lom*)::*bla Φ*(*csrA’-‘lacZ*)*1*(*hyb*) Amp^R^Gm^R^	This study
JEK881	JEK736 *Δ*(*λatt-lom*)::*bla Φ*(*csrA’-‘lacZ*)*1*(*hyb*) Amp^R^Gm^R^Km^R^	This study
JEK1321	JEK710 *Δ*(*λatt-lom*)::*bla Φ*(*uvrY’-‘lacZ*)*1*(*hyb*) Amp^R^Gm^R^	This study
JEK1315	JEK736 *Δ*(*λatt-lom*)::*bla Φ*(*uvrY’-‘lacZ*)*1*(*hyb*) Amp^R^Gm^R^Km^R^	This study
JEK1139	JEK710 Δ*sdiA* Gm^R^Km^R^	This study
JEK1553	JEK710 Δ*H-NS* Gm^R^Cm^R^	This study
JEK1557	JEK710 Δ*H-NS*Δ*nhaR* Gm^R^Cm^R^Km^R^	This study
JEK1598	JEK710 *uvrY*::*uvrY*-3xFlag-Km^R^; Gm^R^	This study
JEK1601	JEK736 *uvrY*::*uvrY*-3xFlag-Km^R^; Gm^R^	This study
Plasmids		
pKD3	Lambda Red mutagenesis plasmid; Cm^R^	[37]
pUC19	Cloning vector; Amp^R^	
pKK223-3	Cloning vector; Amp^R^	Amersham Pharmacia Biotech (Uppsala, Sweden)
pNhaR	*nhaR* in pKK223-3	[22]
pNhaA	*nhaA* cloned under *plac* promoter in pUC19;Amp^R^ (pJEK550)	This study
pG-GFP	Promoter probe vector *gfp*; Amp^R^	[40]
pJEK1224	585 bp of *recA* promoter fragment (-600,-15) cloned into pG-GFP bp fragment	This study
pJEK1263	349 bp of *uvrY* promoter fragment (-358, -9) cloned into pG-GFP	This study
pJEK1270	625 bp of *uvrY* promoter fragment (-634, -9) cloned into pG-GFP	This study
pJEK1272	pJEK1270 with a single deletion in H-NS5 motif	This study
pJEK1275	pJEK1270 with a single substitution in H-NS5 motif	This study
pJEK1292	185 bp of *uvrY* promoter fragment (-196, -9) cloned into pG-GFP	This study
pJEK1295	761 bp of *uvrY* promoter fragment (-770, -9) cloned into pG-GFP	This study
pJEK1301	251 bp of *uvrY* promoter fragment (-260, -9) cloned into pG-GFP	This study
Bacteriophage		
P1 vir	Strictly lytic P1	Carol Gross

### Construction of *lacZ* and GFP reporter fusions

*CsrA*, *csrB*, *csrC*, and *uvrY* transcriptional and translational fusions to *lacZ* were constructed previously [31, 33, 38, 39] and transduced into the JEK710 strain and its derivative mutants. PCR fragments containing *uvrY*, *sdiA*, *recA*, and *lexA* were amplified with specific primer pairs (Table 2), cloned into EcoRI/KpnI sites of the pG-GFP plasmid [40], and introduced into the JEK710 strain and its derivative mutants. GFP activity (A_480-520_) was measured using a BioTek Plate Reader (BioTek) and normalized to the optical density of the culture (A_600_), yielding relative fluorescence units (RFU; A_480-520_/A_600_). Student T-test was used to compare results and check statistical significance.

**Table 2:** Oligonucleotides used in the study.

Primer name	Sequence (5’-3’)	Purpose
prJEK1	/5Biosg/AAA GGC GTA AAG TAG CAC CCA TAG	*csrC* for EMSA.
prJEK40	CAA AGC GGT CGT CTC CGT CAG TC	*csrC* for EMSA.
prJEK6	/5Biosg/TCG ACG AAG ATA GAA TCG TCT T	*csrB* for EMSA.
prJEK7	TAA TCC AAA TAC CCC ATC TGG	*csrB* for EMSA.
prJEK66	AAA AAG CTT GAA ACA TCT GCA TCG ATT CT	*nhaA* for cloning
prJEK67	AAA GGA TCC ACA TGC TCA TTT CTC TCC CTG	*nhaA* for cloning
prJEK123	/5Biosg/GTG GTC ATC AAC AAG TAG AAC G	Reverse for *uvrY* EMSA
prJEK122	GTG GTC ATC AAC AAG TAG AAC G	*uvrY* primer extension 1
prJEK125	CAG TTA TGG TCA CGC CCG TC	*uvrY* primer extension 2
prJEK132	AAA GAA TTC CAG AAA TAG GGA TAA CG	*uvrY* (-9) reverse for cloning into pG-GFP.
prJEK133	AAA GGT ACC GAG CGT GAT ATC GGC AGT GC	*yvrY* (-358) for cloning (pJEK1263) and EMSA.
prJEK135	AAA GGT ACC GCA GCC TGG GTT TCG TCT TC	*uvrY* (-634) for cloning (pJEK1270).
prJEK155	AAA GAA TTC CCG CTA CTG GCT TAA TTT GAT CTC	*uvrY* (-770) for cloning (pJEK1270).
prJEK156	AAA GAA TTC GTT ACA TAT TCA GCG GGC TG	*uvrY* (-260) for cloning (pJEK1301).
prJEK172	CCT CAA CAA ACC ACC CCA ATA TAA GTT TGA GAT TAC TAC AGT GTA GGC TGG AGC TGC TTC	*H-NS* deletion with pKD3
prJEK173	GCC GCT GGC GGG ATT TTA AGC AAG TGC AAT CTA CAA AAG AAT GGG AAT TAG CCA TGG TCC	*H-NS* deletion with pKD3

### Beta-galactosidase assay

Assay for the activity of all *lacZ* reporter fusions was conducted as described earlier [41]ith the following modifications. Cells were permeabilized with 100 μl of chloroform and 50 μl of 0.01% (w/v) SDS and reactions were terminated with 0.5 ml of 1 M Na_2_CO_3_. LacZ activities were presented as Miller units per OD_600_ or protein amounts. Protein estimation was done following the bicinchoninic acid method after precipitation with 10% trichloroacetic acid (TCA). Student T-test was used to compare results and check statistical significance.

### Electrophoretic mobility shift assays (EMSA)

DNA fragments located upstream of the *csrB* (220 bp; pos. -215+5), *csrC* (208 bp; pos. -204+4), and *uvrY* (388 bp pos. -358+30 and 228 bp pos. -198+30) genes were amplified by PCR using specific primer sets (Table 2). Each set contained a single 3’-end commercially biotinylated primer. These PCR products (1fM) and different amounts of purified recombinant NhaR (NhaR-His6) protein [22] were analyzed using the LightShift Chemiluminescent EMSA Kit as described by the manufacturer (Pierce Biotech., Rockford, IL). A previously described 138-bp promoter region of the *pgaABCD* genes [22] was used as a specific control and competitor. Binding reactions were separated on 5% PAGE gels, electrotransferred onto a positively charged nylon membrane (Roche Diagnostics GmbH, Mannheim, Germany), and detected using the Chemiluminescent Nucleic Acid Detection Module (Pierce Biotech., Rockford, IL), according to the protocol. Fluorescent bands were visualized by a ChemiDoc XRS+ Imaging System and analyzed using Quantity One Software (BioRad Lab., Hercules, CA).

### Northern and Western blotting

Total RNA was isolated using the RNAprotect Bacteria Reagent with the RNeasy kit (Qiagen, Valencia, CA) at different time points from bacterial cultures grown with shaking (250 rpm) at 26°C. For blotting, 1.5 μg of total RNA was separated in denaturing conditions on 5% polyacrylamide gels with urea (7 M) and blotted onto positively charged nylon membranes (Roche Diagnostics GmbH, Mannheim, Germany) by electrotransfer for 40 min. The RNA on the membrane was UV crosslinked, stained with methylene blue solution to control transfer efficiency, and developed following the DIG Northern Starter Kit manual (Roche Diagnostics GmbH, Mannheim, Germany) with DIG-labeled RNA probes specific for the *csrB* and *csrC* genes prepared as described previously [42].

The Western blotting protocol was described previously [18]. Bacterial cultures were grown with shaking at 26°C in specified media and cells were harvested at different time points. Cells were mixed with Laemmli sample buffer and lysed by sonication and boiling. Each sample (10 μg protein) was subjected to SDS-PAGE. Proteins were transferred to 0.2-μm PMSF membranes, and NhaR-Flag was detected using anti-Flag monoclonal antibodies as recommended by the manufacturer (Sigma-Aldrich). Quantification analyses were performed using a Syngene GeneTools software.

### Primer extension analysis

Total RNA was prepared from cells grown for 18 h in LB pH 8.4 TAPS at 26°C. Primers prJEK122 and prJEK125 were annealed at positions +30 and -136 relative to the transcript initiation site of *uvrY*, respectively. Primer extension analyses were performed according to the protocol of Wang et al. [27]. Results are shown in Supplementary materials (S1 Fig).

## Results and discussion

### NhaR regulates expression of CsrA system at low temperature in high pH/high salt medium

**Fig 1:**
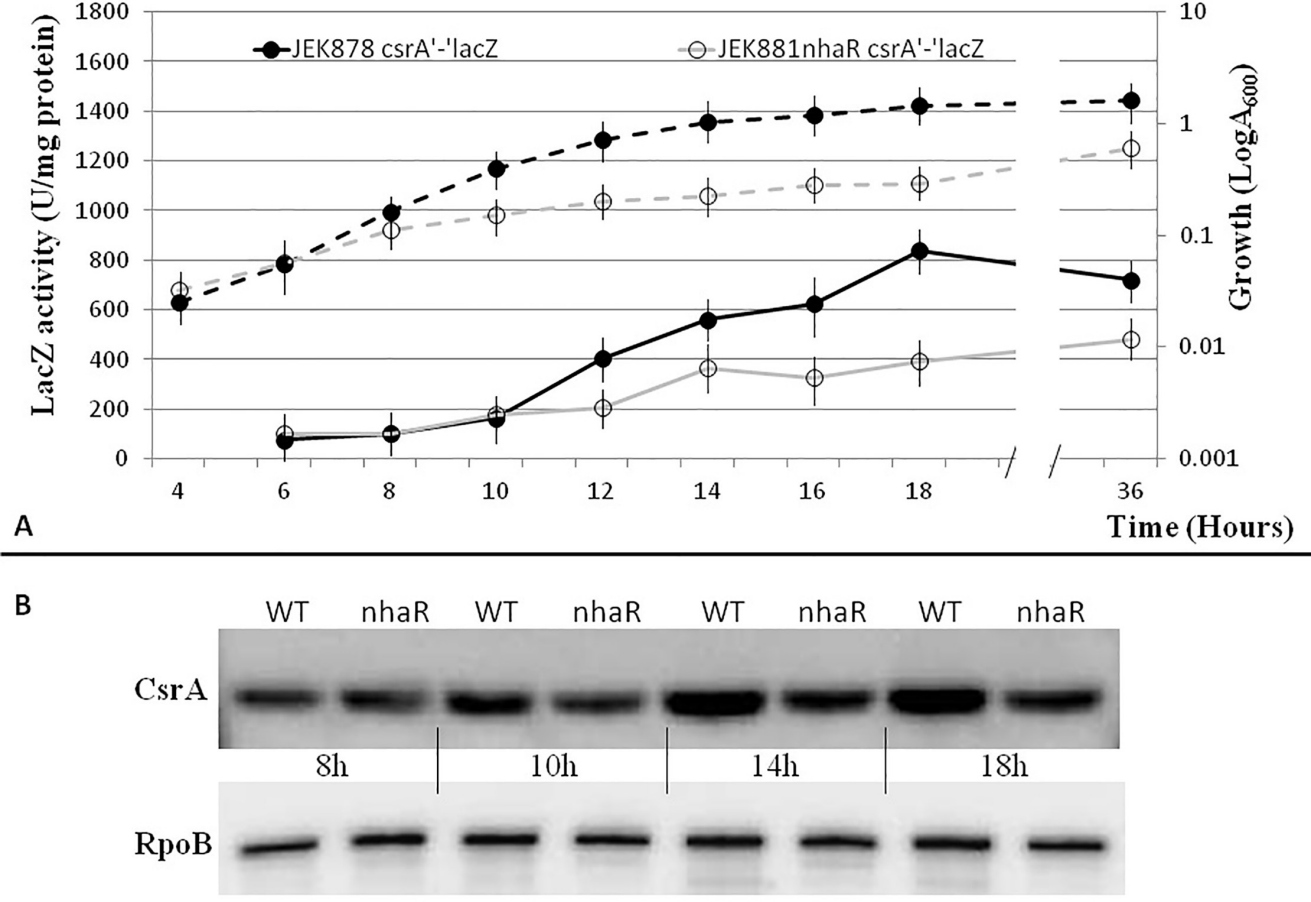
Effect of *nhaR* mutation on *csrA* gene expression. Growth curves (dashed lines) and activity of *csrA’*-*’lacA* fusion (solid lines) in WT and *nhaR* mutant of *E*. *coli* MG1655 grown at 26C in LB pH8.4 TAPS (**A**). Western blot analysis of CsrA protein in WT and *nhaR* mutant (**B**). RpoB was used as loading control. Data shows representative results from 4 experiments.

Previous reports have mentioned that viability of *E*. *coli nhaR* mutant strains is affected in high salt (<0.1 M) and high pH (>8.4) media; however, detailed information about growth conditions was not supplied [13]. In order to study the effect of NhaR on the CsrA system, I defined growth conditions where *nhaR* plays its regulatory role affecting the CsrA system. I used previously constructed *lacZ* gene fusions with *csrA*, *csrB*, and *csrC* genes. These fusions were transduced from the original strains into the *E*. *coli* MG1655*lacZ*^-^ (JEK710) strain and its isogenic *nhaR* mutant (JEK736). As expected, at 37°C in LB pH 7.4 TAPS, the differences between the wild-type (WT) strain and *nhaR* mutant were negligible (data not shown). In LB pH 8.4 TAPS, although the specific *lacZ* activities were slightly lower than in LB pH7.4 medium, no differences between the *nhaR* mutant and the isogenic WT strain were observed (data not shown). A completely different situation was observed when cells were grown in LB pH 8.4 TAPS at low temperature (26°C). The activity of *csrA’*-‘*lacZ* fusion was up to 2.2-fold lower in the *nhaR* mutant strain than in the WT strain (Fig 1A). To confirm these data, I conducted Western blotting analysis of total proteins isolated from the *nhaR* mutant and the isogenic WT strain using CsrA-specific antibodies. Hybridization results confirmed that the amount of CsrA protein in the *nhaR* mutant was lower and the difference level was similar to those for the *lacZ* fusions (Fig 1B). As one could expect, the biggest difference was observed in early stationary phase (in these conditions 14–18 h) when *csrA* gene expression is usually induced. The amount of CsrA in the mutant strain was 1.33 to 1.38 lower than in the WT. The EMS experiment with csrA promoter and NhaR protein showed that the interaction is not specific and can be abolished with a specific (pgaA) and nonspecific (dI-dC) competitors (S2 Fig).

**Fig 2:**
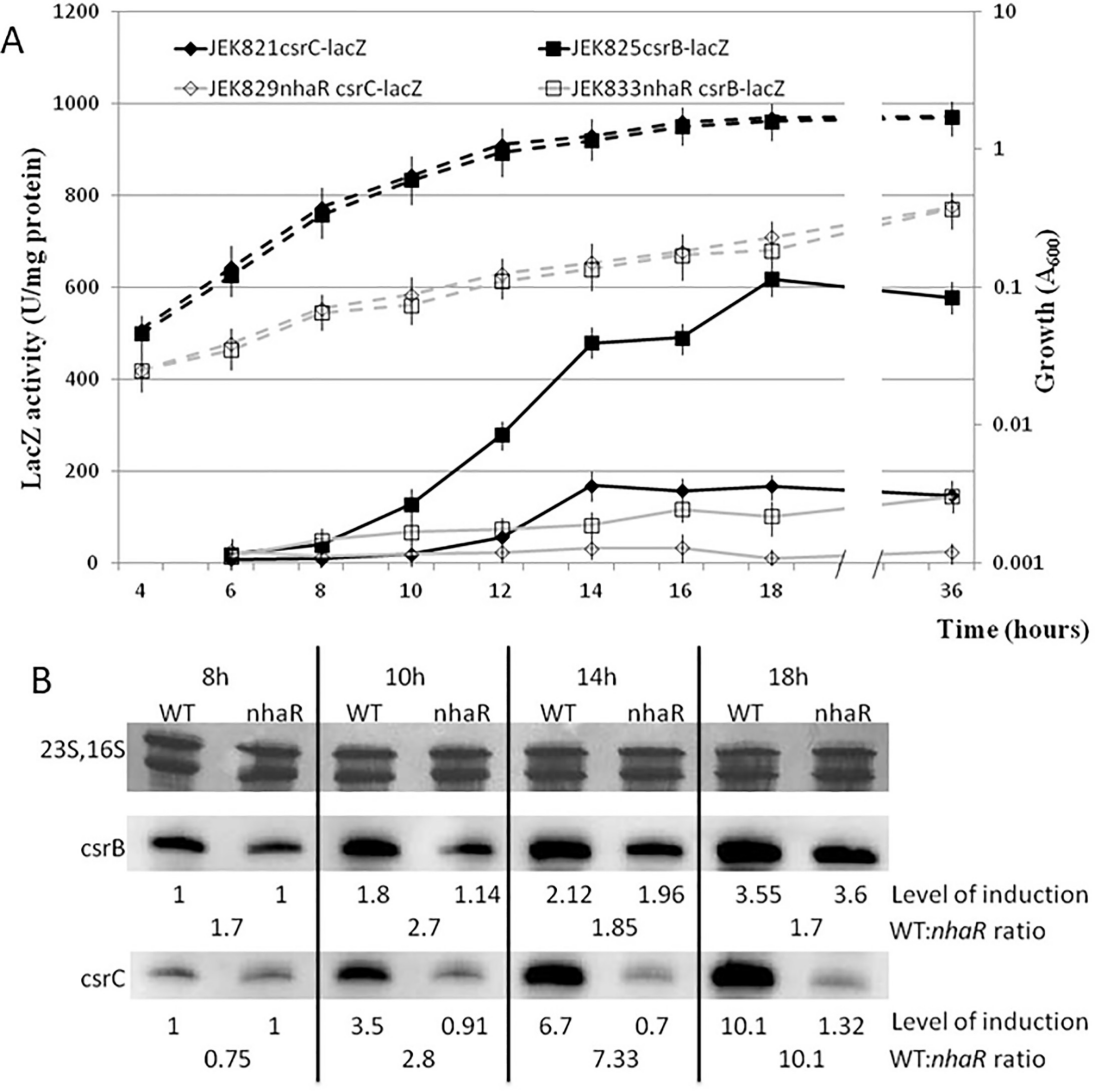
Effect of *nhaR* on *csrB* and *csrC* expression. Growth curves (dashed lines) and activity of *csrB*-*lacZ* and *csrC*-*lacZ* fusions (solid lines) in WT and *nhaR* mutant of *E*. *coli* MG1655, grown at 26°C in LB pH 8.4 TAPS **(A)**. Northern blot analysis of *csrB* and *csrC* transcripts **(B)**. Total RNA was isolated at different time points from the WT and *nhaR* mutant strains grown at 26C in LB pH 8.4 TAPS. Level of induction shows amounts of RNA along the growth curve starting with 1 at 8h; WT:*nhaR* ratio was calculated for each time point. The 16S and 23S rRNA were stained with methylene blue and served as an internal control.

Subsequently, I checked the effect of *nhaR* mutation on two other components of the Csr system: *csrB* and *csrC*. LacZ assay results showed a very strong effect of *nhaR* mutation on expression of both *csrB* and *csrC* genes (Fig 2A). In the WT strain, both the *csrB* and *csrC* genes were induced as cells entered the stationary phase (10–12 h). In the *nhaR* mutant, no induction was observed. The expression of *csrB* was at a very low level while the *csrC* promoter showed almost no activity (Fig 2A). The maximal difference in expression activities between the *nhaR* mutant and isogenic WT strain was 5.2-fold for *csrB* and up to 12-fold for *csrC*.

To confirm these differences, a Northern blot hybridization to total RNA isolated from the *nhaR* mutant and WT strains grown in the same conditions was conducted with *csrB* and *csrC* gene probes. Hybridization results confirmed that *nhaR* is necessary for expression of both small RNAs; however, differences in the *csrB* and *csrC* RNA levels between mutant and WT strains were much lower (max. 2.7- and 10.1-fold for *csrB* and *csrC*, respectively) than those observed in *lacZ* fusions (Fig 2B).

### NhaR affects stability of *csrB* and *csrC* small RNAs

**Fig 3:**
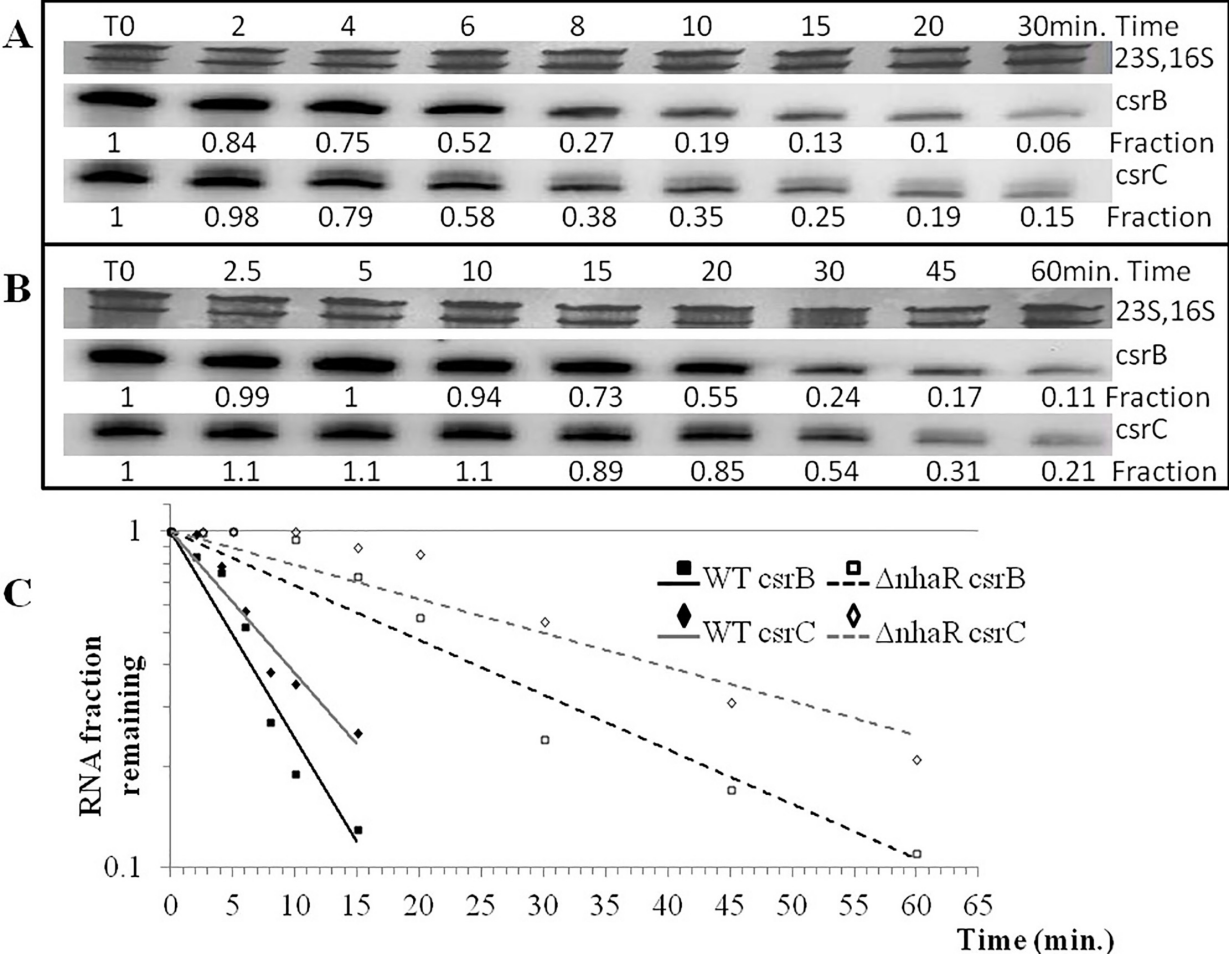
Stability of *csrB* and *csrC* in WT **(A)** and *nhaR* mutant **(B)** of *E*. *coli* MG1655, grown for 18 h at 26°C in LB pH 8.4 TAPS. RNA synthesis was inhibited by rifampicin (300μg/ml) and RNA was isolated at different time points after inhibition. Fraction of remaining RNA at each time point is shown. The 16S and 23S rRNA were stained with methylene blue and served as an internal control. The RNA half-lives were determined from the linear portions of the decay curves **(C)**. The CsrB half-life in isogenic MG1655 (wild type) and *nhaR* mutant strains was 5 and 13 min., respectively. The CsrC half-life in the same strains was 7 and 22 min., respectively.

The difference between the expression levels and the actual RNA levels in the cell can be explained by a higher RNA stability in the *nhaR* mutant strain. To prove this hypothesis, I measured the half-lives of *csrB* and *csrC* RNAs in the *nhaR* and WT strains grown in LB pH 8.4 TAPS at 26°C (Fig 3). The data showed that in the WT strain the turnover for both RNAs was relatively fast (half-lives: 5 and 7 minutes for *csrB* and *csrC*, respectively), while in the *nhaR* mutant strain those RNAs were much more stable with half-lives 2.6 and 3.14 times longer for *csrB* and *csrC*, respectively, than in the WT strain. These differences in small RNA stability explained the observed differences between the transcriptional activity measured by *lacZ* fusions and the actual RNA levels shown by Northern hybridization.

To check if NhaR directly activates transcription of *csrB* and *csrC* genes, I used an electrophoretic mobility shift assay (EMSA; See Methods section). Obtained results suggested that NhaR does not interact directly with the *csrB* and *csrC* promoters (data not shown).

### NhaR regulates *uvrY* expression

**Fig 4:**
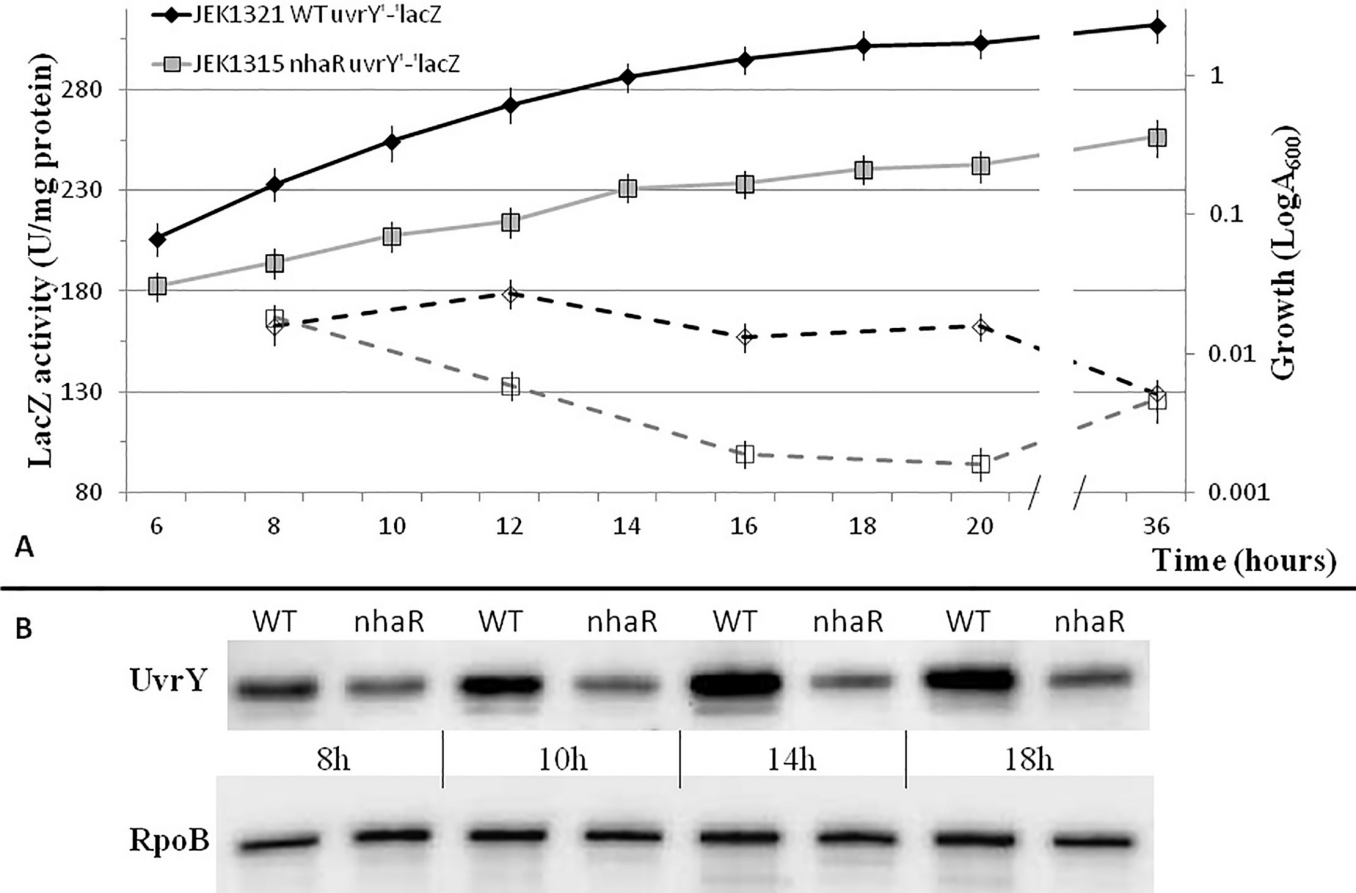
Effect of *nhaR* mutation on *uvrY* gene expression. Growth curves (solid lines) and activity of *uvrY’*-*’lacA* fusion (dashed lines) in WT (JEK1321) and *nhaR* mutant (JEK1315) of *E*. *coli* MG1655 grown at 26C in LB pH8.4 TAPS (**A**). Western blot analysis of the UvrY-Flag protein in WT and *nhaR* mutant (**B**). RpoB was used as loading control. Data shows representative results from 3 experiments.

Expression of *csrB* and *csrC* RNAs depends on the presence of the TCS response regulator UvrY [33]; therefore, I analyzed if mutation in the *nhaR* gene somehow affects expression of the *uvrY* gene. The previously constructed translational *uvrY’*-‘*lacZ* fusion (KSY0009) was transferred into *E*. *coli* MG1655 JEK710 and its isogenic *nhaR* mutant strains. A time course experiment showed that the expression of *uvrY* gene was up to 1.7-fold lower in the mutant strain in early stationary phase (Fig 4A).

To confirm these results, I analyzed the actual level of UvrY protein. The chromosomal *uvrY* gene was modified *in situ* such that it produced a protein containing a 3xFlag epitope tag at the C terminus. Western immunoblotting studies with an anti-Flag monoclonal antibody revealed that the UvrY-Flag protein level was higher (up to 7-fold) in the WT than in the isogenic *nhaR* mutant (Fig 4B).

### IS*5* insertion in the *sdiA*-*uvrY* intergenic region abolishes the effect of *nhaR* mutation

**Fig 5:**
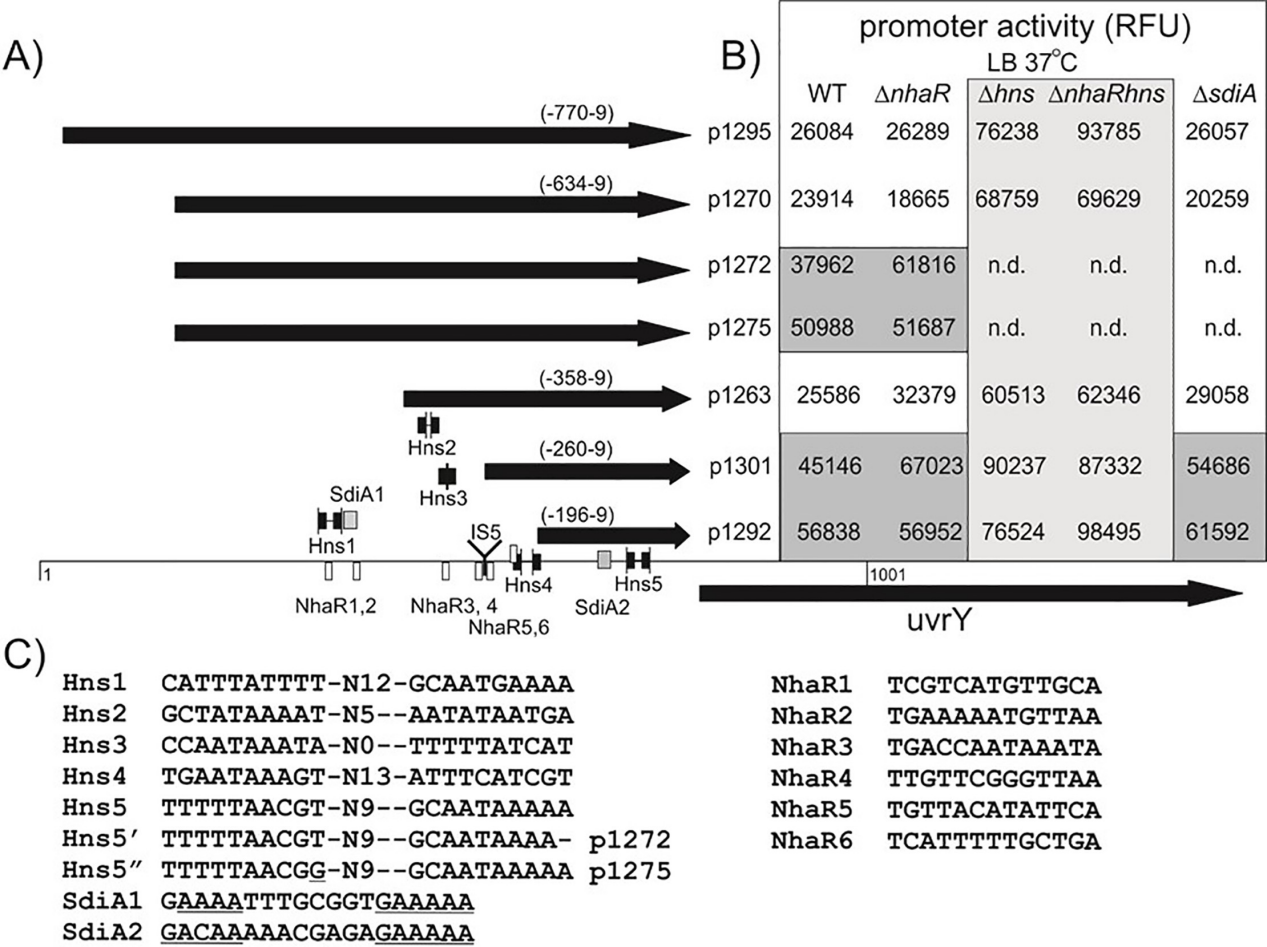
Analysis of *uvrY* promoter region. Fragments cloned into pG-GFP reporter plasmid are represented by arrows (**A**). Predicted binding sites for H-NS, SdiA and NhaR are marked as linked black arrows, gray and white boxes, respectively. Expression activity of each clone in a given strain in standard LB (18h cultures at 37°C) is presented in the table (**B**). DNA sequence for each binding site including two mutations in H-NS5, is shown. Conserved motifs in SdiA sites are underlined (**C**).

During my experiments with *csrC*-*lacZ* gene fusions and Northern hybridizations, I noticed that some of my clones behaved differently–showing no effect of *nhaR* mutation on both LacZ activity and the *csrB* and *csrC* levels (data not shown). Amplification of the *sdiA*-*uvrY* intergenic region revealed that the DNA fragment amplified from those atypical strains was ~1.3 kb bigger than in the WT *E*. *coli* MG1655 strain (data not shown). The DNA sequence analysis showed an insertion of IS*5* element in that region, 260 bp upstream of the *uvrY* start codon (Fig 5).

### *In silico* analysis of regulatory motifs in *uvrY* promoter region

Back in the mid 1980’s when the UvrY gene was originally described, three promoters were identified upstream of the *uvrY* gene: one promoter in front of the current *sdiA* gene (P1), and two promoters (P2a and P2b) in front of the *uvrY* gene [43]. My *in silico* and primer extension analyses revealed the possible presence of additional promoter sequences in front of the *uvrY* gene (See S1 Fig). Primer extension analysis showed that the only difference between the WT and *nhaR* mutant strains was lack of a single extension product (T-542) in the mutant strain (See S1 Fig).

The DNA sequence motif that is recognized by the NhaR protein is not well characterized. According to the PRODORIC Database [44], the consensus position weight matrix constructed for the NhaR binding site contained 11 nucleotides 5’- TcgtaAaAAac-3’. I noticed that this motif was not correct as all LysR-type regulators recognize a consensus sequence (T-N_11_-A) [45]. Using D-MATRIX software [46] and available sequence data [43, 47], I constructed a new *nhaR* consensus sequence 5’–TCgaAAAAatCtA-3’ and identified at least 6 hypothetical NhaR binding sites in front of *uvrY* gene (Fig 5). I noticed that three of the NhaR binding sites were located immediately upstream of the T-542 transcription start, which was not detected in the *nhaR* mutant strain (see above).

As some reports showed that *uvrY* gene expression is regulated by the SdiA protein, I also searched for the presence of a DNA motif that might be responsible for binding of that regulator. SdiA belongs to the LuxR family and its binding site was determined as 5′-AAAAGNNNNNNNNGAAAA-3′ [48]. Searching for similar motifs, I was able to identify two regions with high similarity. One of them was located 102 bp upstream of the *uvrY* start codon while the second was located further upstream (-415 bp) (Fig 5).

While running the virtual footprint analysis, I also noticed a high number of motifs responsible for binding of the H-NS protein. As NhaR is known to interact with H-NS protein, as in the case of *nhaAR* regulation, I decided to have a closer look at the location of those sites. Ten H-NS binding sites were predicted in total. Analysis of detailed location revealed that these sites form a kind of “dyad” structures with some of them oriented toward each other (H-NS 2, 3, 4) and some heading in opposite directions (H-NS1, 5) (Fig 5). I noticed that three out of five hypothetical binding sites for the H-NS regulator overlapped with the predicted NhaR sites. Also, the distal SdiA binding motif overlapped with the NhaR binding site (Fig 5), suggesting that all three regulators play a role in the regulation of *uvrY* expression.

### NhaR interacts with H-NS and SdiA in regulation of the *uvrY* promoter

To analyze the effect of these three regulators, I amplified a set of nested DNA fragments with a downstream primer located at pos. -9 in relation to the *uvrY* start codon and different upper primers. Obtained amplicons were cloned in front of a promoterless *gfp* gene in a pG-GFP promoter probe vector [40] resulting in plasmids pJEK1295 (-770;-9bp in relation to *uvrY* start point), pJEK1270, pJEK1272, pJEK1275 (all -634;-9bp), pJEK1263 (-358;-9bp), pJEK1301 (-260;-9bp), and pJEK1295 (-196;-9bp in relation to *uvrY* start point) (Fig 5). All clones were sequenced to confirm DNA sequence identity. When fragment (-634,-9) was cloned, I noticed that two clones showed a stronger fluorescence than others (pJEK1272 and p1275). DNA sequence analysis revealed the presence of a single-base deletion (pJEK1272) and a single-base substitution (pJEK1275) within the H-NS5 region (Fig 5). These two, together with other plasmids, were transferred into *E*. *coli* MG1655 (JEK710) and its isogenic *nhaR*, *H-NS*, *nhaRH-NS*, and *sdiA* deletion mutants. Promoter activity was measured as fluorescence (A_480-520_) per culture density (A_600_) and expressed as relative fluorescence units (RFU). At 37°C in standard LB medium, the basic *uvrY* promoter activity was 25,400±4,100 RFU and was observed for pJEK1295, p1270, and p1263 in the WT, Δ*nhaR*, and Δ*sdiA* strains. As plasmid pJEK1263 did not contain NhaR1, NhaR2, SdiA1, and H-NS1 sites, it seemed that those sites are not important for the expression of *uvrY* promoter under these conditions. Plasmid pJEK1301 contained a 251-bp fragment that lacks H-NS2, H-NS3, as well as NhaR3 and NhaR4 binding sites and was designed specially to imitate the effect of *IS5* insertion (Fig 5). Its activity in the WT strain was 1.8 times higher than longer fragments (P = 0.0012). Also, in the *nhaR* and *sdiA* mutants, the activity of the shorter fragment was 2.64 and 2.15 higher than the p1295 fragment, respectively. The shortest fragment pJEK1292 (196 upstream of the *uvrY* start codon) contained only H-NS5 and SdiA2 motifs. Its specific activity was again ~12% higher than pJEK1301 in the WT and *sdiA* mutant strains and 15% lower in the *nhaR* mutant (Fig 5), suggesting that the presence of NhaR and both NhaR5 and NhaR6 binding sites slightly activates expression of the *uvrY* promoter. In *E*. *coli H-NS* and *H-NS*/*nhaR* double-deletion mutants, the activity of all reporter plasmids was much (2.4- to 3.9-fold) higher than in the corresponding WT strain and these results were extremely statistically significant (P = 0.0007 for H-NS and P = 0.001 for the double mutant) (Fig 5B). Also, plasmids pJEK1272 and p1275, which carry single-nucleotide mutations in the H-NS5 site, showed 1.5- to 2.4-fold increases in activity, respectively, compared to the WT strain (P = 0.0013 and P = 0.0012, respectively) (Fig 5). All these data show strong statistical significance and strongly suggest that the H-NS protein is involved in inhibition of the *uvrY* promoter.

**Fig 6:**
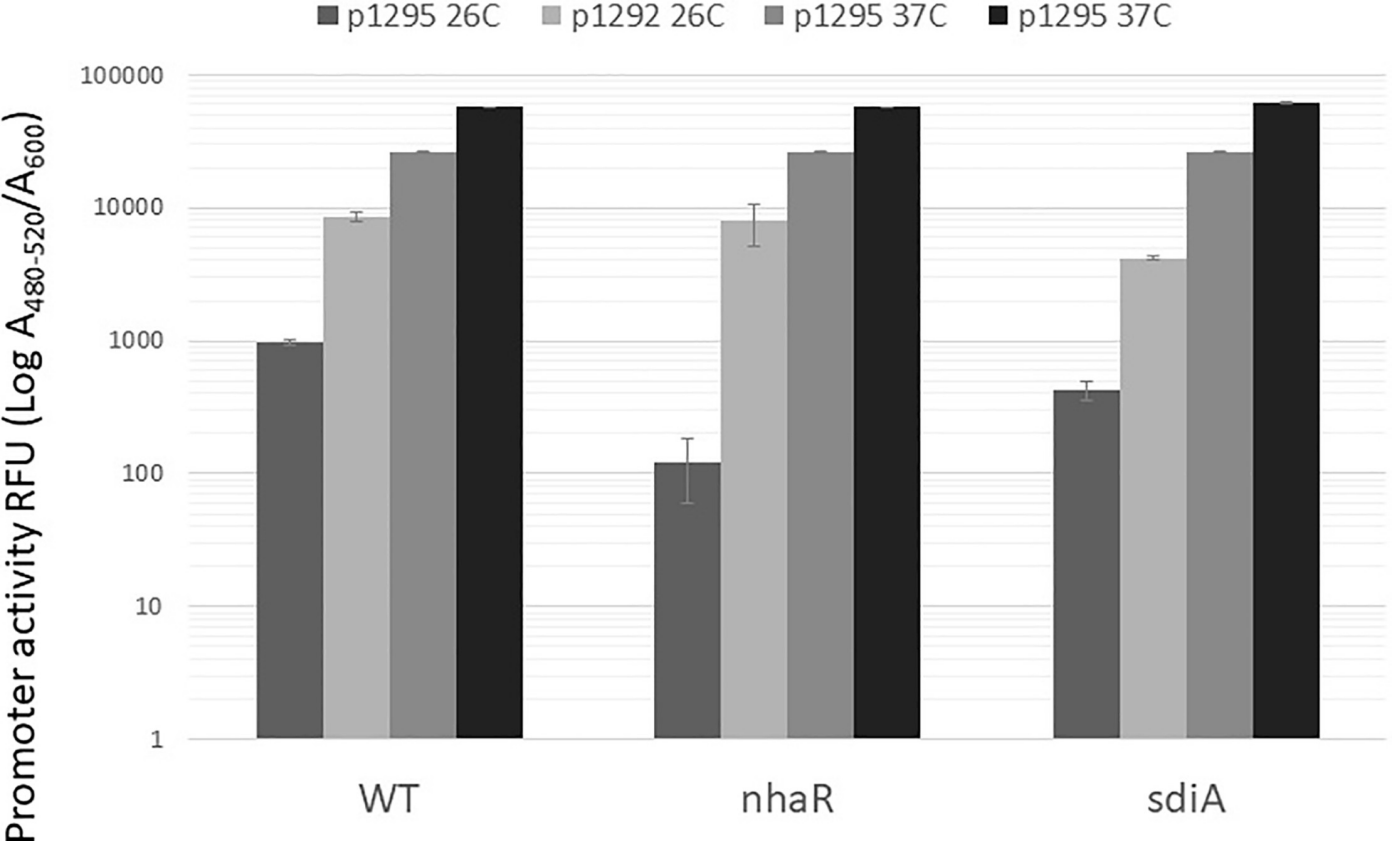
Transcriptional activity of selected fragments from the *uvrY* promoter region in stress conditions. *E*. *coli* MG1655 (JEK710) and its isogenic *nhaR* and *sdiA* deletion mutants with reporter plasmids were grown for 18 h at 26°C in LB pH8.4 TAPS. Fluorescence and cell density were measured to calculate promoter activity in RFU. Data shows representative results from 3 experiments.

I also analyzed *uvrY* promoter activity under the conditions where I saw the biggest effect of *nhaR* mutation on the *csrA* system (18 h, 26°C LB pH 8.4 TAPS). Results showed that under these conditions the activity of the *uvrY* promoter was much lower than at 37°C (Fig 6). For the longest fragment, which contained the whole intergenic region (pJEK1295), that difference was more than 26-fold, while for the shortest one (pJEK1292), that difference was 6.6-fold, in the WT strain. The major observation was that in both *nhaR* and *sdiA* mutants, the activities of the longer fragment were reduced 8 and 2.3 times compared to the WT strain (Fig 6). Both results were statistically significant with P = 0.0004 and P = 0.0024, respectively. That 8-fold difference in *uvrY* promoter activity between the WT and *nhaR* mutant was closer to the effect observed in NhaR Western blot analysis where the difference was much higher (7-fold) than between the corresponding *uvrY’*-‘*lacZ* fusions (Fig 4). In the case of the shorter fragment, mutation in the *nhaR* gene did not affect its activity (P = 0.25) while *sdiA* mutation reduced its activity 2-fold (P = 0.0048). As that fragment contains the second SdiA binding site (SdiA2, Fig 5), it was not surprising that the lack of that regulator reduced its expression activity. Unfortunately, I was not able to analyze *uvrY* promoter activity in the *H-NS* mutant strain under these conditions using the pG-GFP constructs. Antibiotic selection and cost of plasmid-expressing GFP protein together with the *H-NS* mutation made the strain unable to grow in LB pH 8.4 TAPS at 26°C.

### Mapping of the *sdiA*-*uvrY* intergenic region containing the regulatory signals recognized by NhaR

**Fig 7:**
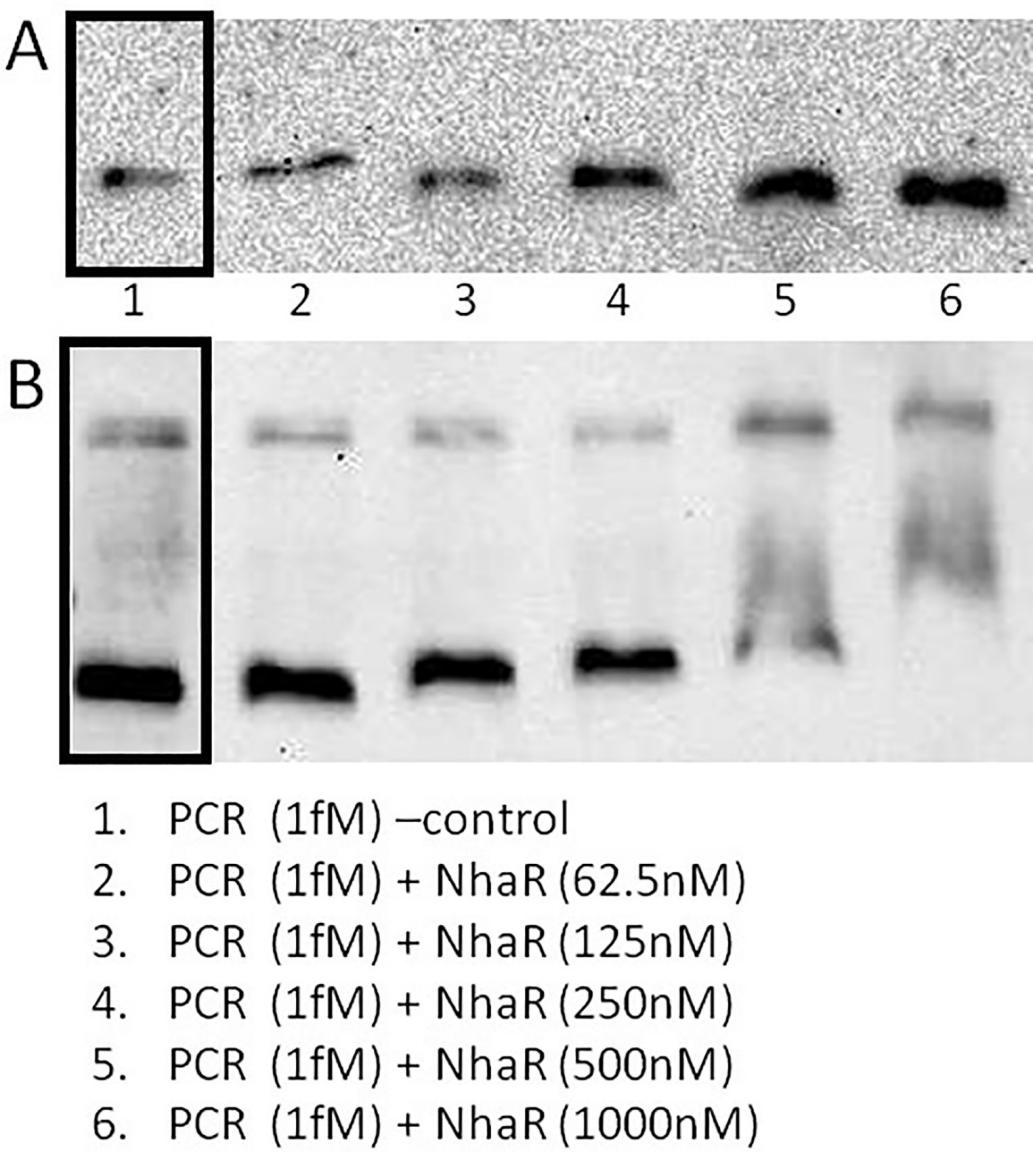
Mapping of the DNA region containing the *cis*-regulatory elements of *uvrY* recognized by NhaR. PCR- amplified fragments from pJEK1292 (**A**) and p1263 (**B**) were incubated with increasing amounts of NhaR protein for 20 min. at room temperature in a buffer containing 10mM Tris pH7.5, 50mM KCl, 1mM DTT, 5mM MgCl_2_ and 2.5% glycerol and detected by immunoblotting. In “B” lower concentrations of NhaR were removed and lines 2–6 were moved left next to the control line 1. Boxed lines–controls without NhaR.

Two fragments pJEK1295 and p1292 showed different sensitivity to *nhaR* mutation (Fig 6). Although *in silico* analysis showed that all hypothetical NhaR binding sites were located further upstream, I decided to confirm if that DNA region contains the *cis*-elements recognized by NhaR. I PCR-amplified sequences from pJEK1292 and p1263 that contained four predicted NhaR sites (NhaR3-6, Fig 5) using a DIG-labeled primer. Each fragment was tested for binding to the purified NhaR-His tagged protein in a DNA gel retardation assay (Fig 7). It was previously shown that the purified protein binds specifically to the *pgaA* gene promoter [22]. My data showed that in fact the smaller PCR fragment did not react with increased NhaR protein concentrations (Fig 7A), while the longer amplicon was shifted when 125 and 250 nM NhaR was added, with even bigger shifts at higher protein concentrations (Fig 7B). That experiment confirmed that in fact there were no sites recognized by the NhaR protein in pJEK1292 and predicted sites located further upstream (pJEK1263) were responsible for NhaR regulation.

**Fig 8:**
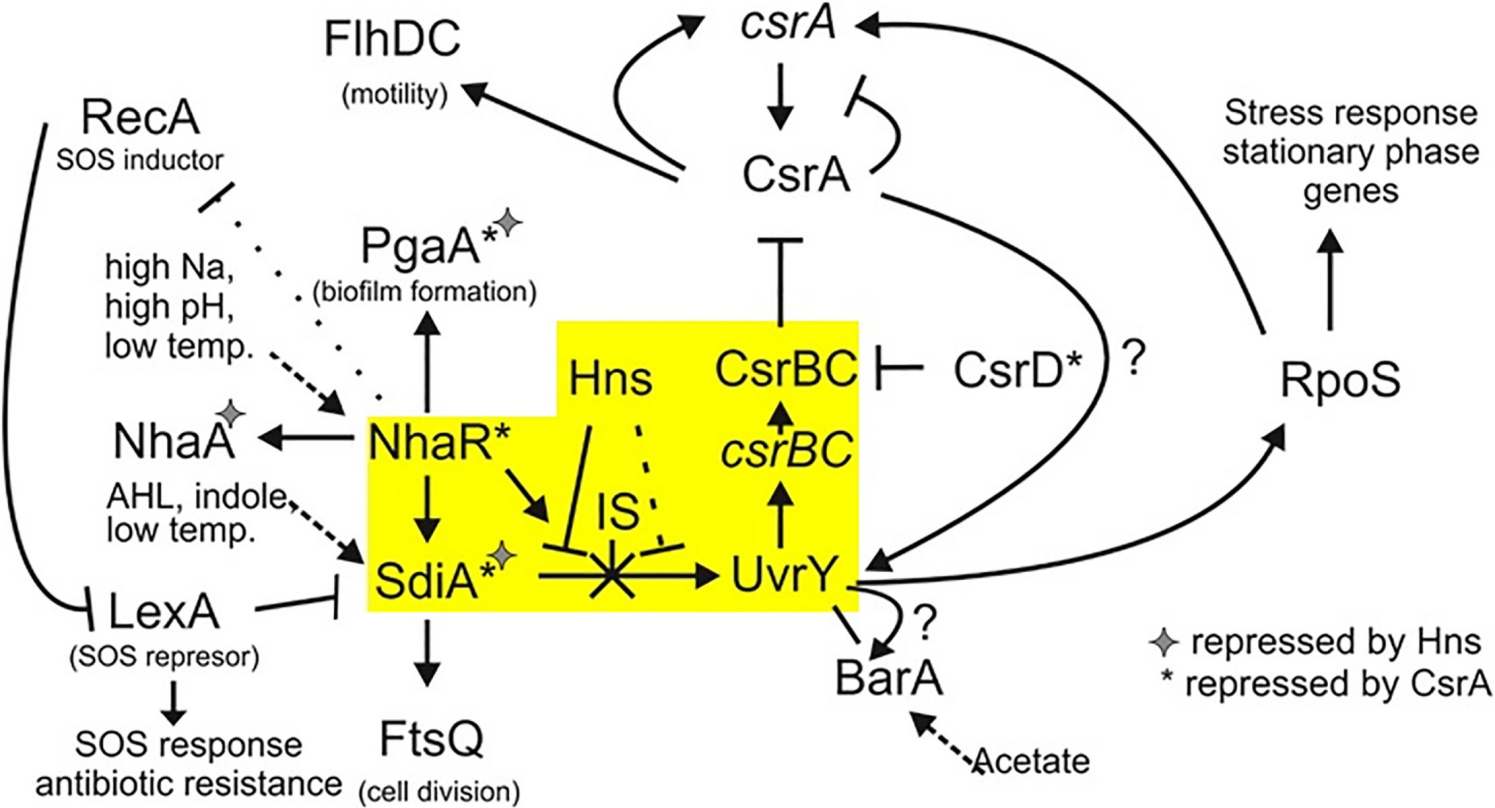
Model of interactions between NhaR, SdiA and H-NS affecting the Csr system activity. Possible indirect effect of NhaR on RecA is shown as a dotted line. Transcriptional and posttranscriptional gene inductors are shown in the case of *nhaR*, *sdiA* and BarA. A dotted line in the case of H-NS represents a possible weak repression by reaction with the H-NS5 binding site. The yellow box highlights the interactions analyzed in this work.

Based on my experimental data as well as available literature, I drew a broader model of interactions involving NhaR, H-NS, and SdiA that affect the Csr regulatory system (Fig 8). As the UvrY/CsrA branch of my model has been recently reviewed [49], here I focus only on the part involving NhaR regulation. The NhaR has been shown previously to regulate its own operon (*nhaAR*) [13], the *pgaABCD* operon [22], as well as the *osmC* gene [20] (omitted in my model). Here I showed that NhaR under specific stress conditions is necessary for expression of the *uvrY* gene. It has been shown previously that UvrY activity was increased under the alkaline conditions [34]; however, a detailed mechanism of that regulation was not revealed. Here I showed that that mechanism depended on NhaR and involved interaction with H-NS and SdiA, as I showed that regulation is extremely important at low temperature when both H-NS and SdiA are induced [24]. In addition, under alkaline conditions, the activity of amino acid deaminases including TnpA is induced resulting in indole production [1] which can act as a quorum-sensing particle activating SdiA [50]. The effect of indole and SdiA on biofilm formation has been published [50]; however, I could not confirm those data using my *E*. *coli* strains (data not shown). I noticed that in some laboratory strains the regulation of the *uvrY* gene was completely destroyed by the presence of the IS*5* insertion sequence in the *uvrY* promoter. That randomly occurring transposition into a *nhaR* “hot spot” can explain observed differences in response to indole.

However, the NhaR is necessary for the regulation of *uvrY*, it seems that the H-NS protein is the major player in that system. An effect of *H-NS* mutation on β-1,6-GlcNAc (PGA) synthesis and biofilm formation has been checked in our lab previously, but only weak changes were observed [22]. As the H-NS protein shows a domain structure, it has been shown that some mutations do not affect its activity [51]. I noticed that in the previous mutant a Tn10 insertion was located near the 3’end of the gene and could explain the data. When a complete deletion *H-NS* mutant was tested in a standard 96-well plate experiment at 26°C, an almost 4.7-fold increase in biofilm formation with respect to the WT strain was observed (data not shown).

In the “omics” era, it is relatively easy to study gene expression in different environmental-stress conditions. Recent work using an Integrative FourD-omic approach (INFO) [52] and the next generation sequencing of immunoprecipitated DNA fragments (CLIP-seq) [53] revealed and confirmed a global role of the CsrA system in gene regulation. Despite that, detailed mechanisms of regulation and interactions affecting expression of specific genes or pathways still have to be studied at the molecular level. My current work may serve as an introduction to more detailed molecular studies of interactions among the NhaR, H-NS, and SdiA regulators.

## Supporting information

S1 Fig Primer extension analysis of the *uvrY* promoter region using total RNA isolated from the WT strain (PE, PE1) and *nhaR* mutant strains (PE2): A- primer JEK122 (+30), B-primer JEK125 (-136). DNA sequence upstream the *uvrY* gene (C); primers are underlined, primer extension products starts are marked as capital letters, predicted binding sites for Hns underlined italics with arrows showing orientation, SdiA and NhaR binding sites are bold and bold underlined respectively; IS5 insertion site is marked by a triangle.
(TIFF)

S2 Fig Interactions between the csrA promoter region and NhaR protein analyzed by EMS. (TIFF)
