# Supplementary figures and images for: Regulatory loop between the CsrA system and NhaR, a high salt/high pH regulator
Source: PLoS One. 2018 Dec 27;13(12):e0209554. doi: 10.1371/journal.pone.0209554 (PMC6307784; doi:10.1371/journal.pone.0209554)

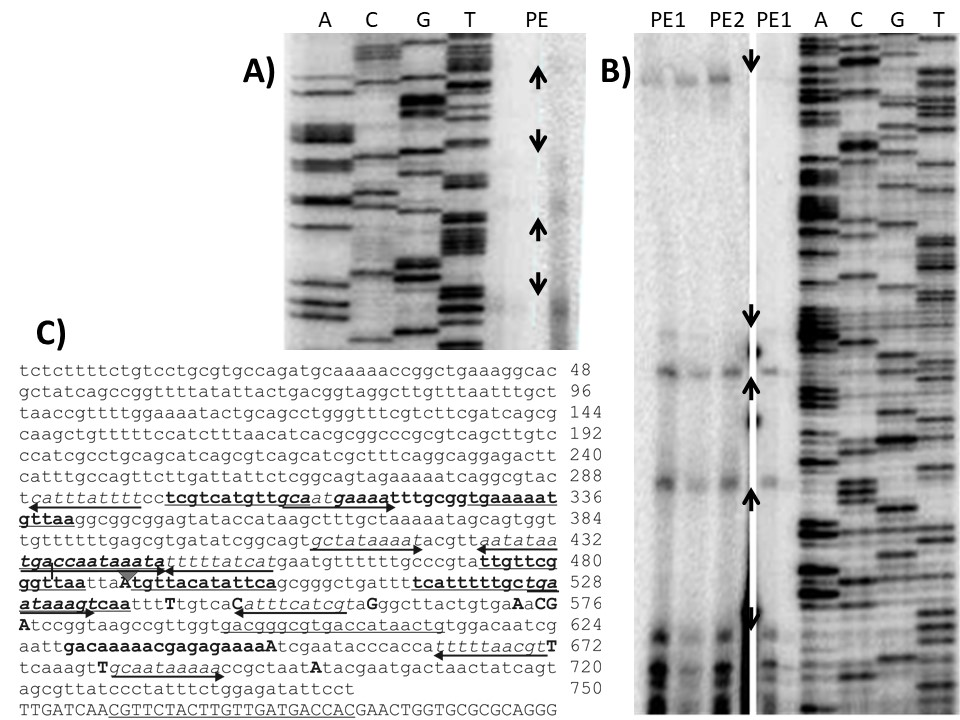

Supplement: S1 Fig — DNA sequence upstream the uvrY gene (C); primers are underlined, primer extension products starts are marked as capital letters, predicted binding sites for Hns underlined italics with arrows showing orientation, SdiA and NhaR binding sites are bold and bold underlined respectively; IS5 insertion site is marked by a triangle. (TIFF) [file pone.0209554.s001.tiff]

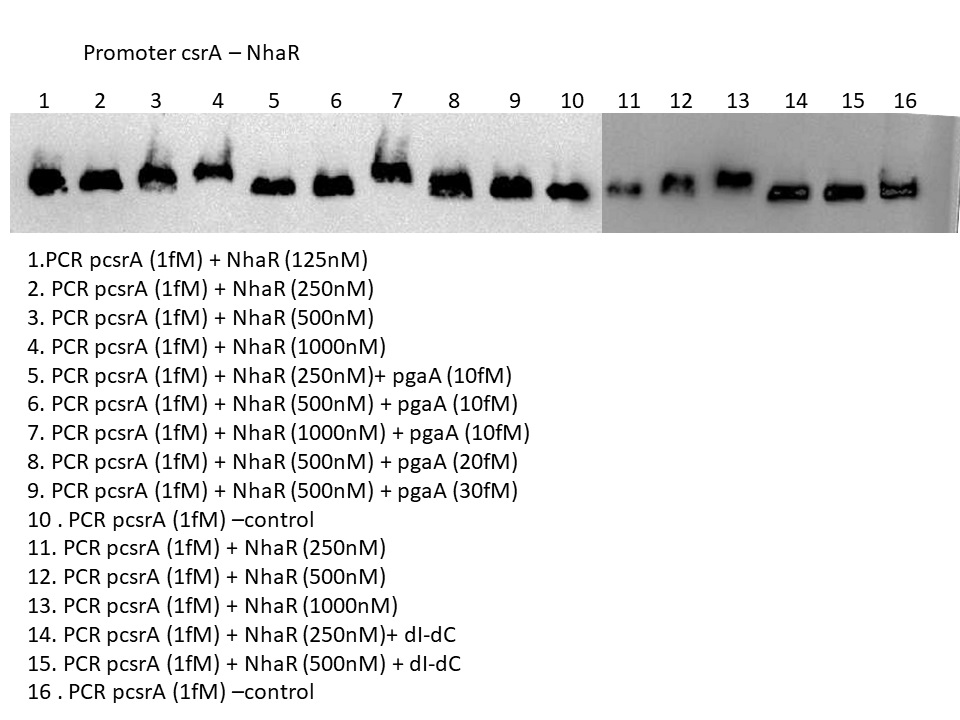

Supplement: S2 Fig — (TIFF) [file pone.0209554.s002.tiff]
